# DNA barcoding is currently unreliable for species identification in most crayfishes

**DOI:** 10.1002/ece3.70050

**Published:** 2024-07-21

**Authors:** Patrick F. Allison, Emily T. Pickich, Zanethia C. Barnett, Ryan C. Garrick

**Affiliations:** ^1^ Department of Biology University of Mississippi University Mississippi USA; ^2^ Southern Research Station USDA Forest Service, Center for Bottomland Hardwoods Research Clemson South Carolina USA

**Keywords:** cryptic species, decapod, global barcoding gap, local barcoding gap, species delimitation, species identification

## Abstract

DNA barcoding is commonly used for species identification. Despite this, there has not been a comprehensive assessment of the utility of DNA barcoding in crayfishes (Decapoda: Astacidea). Here we examined the extent to which local barcoding gaps (used for species identification) and global barcoding gaps (used for species discovery) exist among crayfishes, and whether global gaps met a previously suggested 10× threshold (mean interspecific difference being 10× larger than mean intra specific difference). We examined barcoding gaps using publicly available mitochondrial COI sequence data from the National Center for Biotechnology Information's nucleotide database. We created two versions of the COI datasets used for downstream analyses: one focused on the number of unique haplotypes (*N*_H_) per species, and another that focused on total number of sequences (*N*_S_; i.e., including redundant haplotypes) per species. A total of 81 species were included, with 58 species and five genera from the family Cambaridae and 23 species from three genera from the family Parastacidae. Local barcoding gaps were present in only 30 species (20 Cambaridae and 10 Parastacidae species). We detected global barcoding gaps in only four genera (*Cambarus*, *Cherax*, *Euastacus*, and *Tenuibranchiurus*), which were all below (4.2× to 5.2×) the previously suggested 10× threshold. We propose that a ~5× threshold would be a more appropriate working hypothesis for species discovery. While the *N*_H_ and *N*_S_ datasets yielded largely similar results, there were some discrepant inferences. To understand why some species lacked a local barcoding gap, we performed species delimitation analyses for each genus using the *N*_H_ dataset. These results suggest that current taxonomy in crayfishes may be inadequate for the majority of examined species, and that even species with local barcoding gaps present may be in need of taxonomic revisions. Currently, the utility of DNA barcoding for species identification and discovery in crayfish is quite limited, and caution should be exercised when mitochondrial‐based approaches are used in place of taxonomic expertise. Assessment of the evidence for local and global barcoding gaps is important for understanding the reliability of molecular species identification and discovery, but outcomes are dependent on the current state of taxonomy. As this improves (e.g., via resolving species complexes, possibly elevating some subspecies to the species‐level status, and redressing specimen misidentifications in natural history and other collections), so too will the utility of DNA barcoding.

## Graphical abstract

In this paper, we assembled publicly available mitochondrial DNA (COI gene) sequence data from NCBI's nucleotide database and subsequently compared outcomes from analyses of two different types of COI dataset: one composed only of unique haplotypes for each species, and another that included redundant haplotypes. We found that only 30 of the 81 crayfish species examined can be reliably identified by DNA barcoding. Furthermore, only four of the eight genera that we examined had a global barcoding gap, indicating that many new species may remain undiscovered if solely relying on this approach to uncover cryptic diversity.

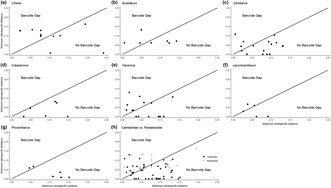

## 1 INTRODUCTION

Species identification is traditionally done using taxonomic keys based on diagnostic morphological characters, but the advent of widely available molecular tools has created opportunities for additional identification methods to be used. Hebert et al. (2003) proposed a DNA barcoding approach whereby a segment of the mitochondrial cytochrome *c* oxidase I (COI) gene is the basis for species identification. Using a library of COI sequences linked to voucher specimens identified by expert taxonomists, barcoding was proposed to be a reliable, cost‐effective, and accessible method applicable to all animals (Hebert et al., 2003). To examine its effectiveness, studies have assessed evidence for barcoding “gaps” which reflect the existence of consistently lower levels of COI sequence differences within species, compared to the levels seen among species (Meyer & Paulay, 2005). The “local” barcoding gap is used for species identification, and this is based on comparing the maximum intraspecific genetic distance among members of a given focal species with the minimum interspecific genetic distance among a set of congeneric species (Collins & Cruickshank, 2013; Meyer & Paulay, 2005). When a local barcoding gap exists, the focal species is considered sufficiently genetically distinct from close relatives that it can be reliably identified based on COI sequence data alone. Conversely, the “global” barcoding gap is used for discovery of morphologically cryptic species, and this is assessed by comparing frequency distributions of mean intra‐ versus interspecific pairwise genetic distances from a pool of named (and possibly as‐yet unnamed) species (Collins & Cruickshank, 2013; Hebert et al., 2004). When a global barcoding gap is present (i.e., when there is no overlap between the two aforementioned frequency distributions), genetically divergent specimens can be flagged as provisional species. This has typically been applied using a threshold‐based approach, such as where mean interspecific differences are assumed or required to be 10× larger than the mean intraspecific differences (i.e., the “10× rule”; Hebert et al., 2004).

DNA barcoding has been broadly applied for species identification and discovery, either alone or in combination with other data. For example, COI sequence differences and morphological characters have been combined to identify butterflies that are difficult to morphologically distinguish (Emery et al., 2009). Barcoding approaches have also led to extensions of the known range of species that are difficult to identify, such as marine gastropods (e.g., *Engina alveolata*; Ran et al., 2020) and giant clams (Velkeneers et al., 2022). Indeed, DNA barcoding has accelerated the discovery of new species, including fishes (Esmaeili et al., 2020), giant clams (Liu et al., 2020), and amphipods (Bradford et al., 2010; Mohrbeck et al., 2021). Molecular taxonomic identification of DNA sampled from bulk environmental collections (eDNA), such as soil and water, also relies on DNA barcoding and is a promising method for detecting rare or newly invasive species (Senapati et al., 2019). DNA barcoding has also enabled pre‐ and post‐mortem analyses of invertebrate diets by amplifying COI from regurgitates (Waldner & Traugott, 2012) and gut contents (Blankenship & Yayanos, 2005). Other ecological applications have included examining stream invertebrate abundance in connection with nutrient and sediment levels (Macher et al., 2016). Further, biodiversity assessments can make use of “DNA metabarcoding” to identify multiple species from bulk samples of organisms or environmental samples (Taberlet et al., 2012).

While DNA barcoding has been used to identify species and discover cryptic diversity within species, there are also some concerns. Hebert et al. (2004) acknowledged that the 10× rule would overlook species involved in hybridization, or sister species that diverged recently. Indeed, Hickerson et al. (2006) showed that single‐gene thresholds can only reliably discover new species with low (i.e., <10%) error rates when genetic isolation occurred >4 million generations ago. DNA barcoding is also much less effective when applied to groups with outdated taxonomy, or where geographic sampling is limited relative to the range of species, or when taxon sampling is incomplete (e.g., sister species are not represented; Meyer & Paulay, 2005). Generally speaking, the information carried by mitochondrial DNA sequence data alone has limitations when inferring species boundaries, owing to the confounding impacts of incomplete lineage sorting, undetected male‐mediated gene flow, and/or hybridization and introgression (Moritz & Cicero, 2004). Additionally, nuclear‐mitochondrial pseudogenes (i.e., duplicated and non‐functional regions of the mitochondrial genome that have integrated into the nuclear genome) can be co‐amplified or preferentially amplified via polymerase chain reaction, either preventing clean COI sequences from being obtained or leading to erroneous inferences about species identification and relationships (Bensasson et al., 2001; Buhay, 2009; Song et al., 2008). Given the potential limitations of DNA barcoding, it is valuable to evaluate its effectiveness separately for different taxonomic groups.

Taxa that are difficult to identify based on morphology, such as many invertebrates, stand to benefit most from DNA barcoding, both in terms of species identification and species discovery. Crayfish are a diverse group of freshwater invertebrates found on every continent except Africa and Antarctica (Crandall & de Grave, 2017; Taylor et al., 2007). Identifying crayfish based on morphology is challenging, and for some families such as Cambaridae, a breeding form male (form I) is required for accurate species identification (Hobbs, 1972). Given the potential unavailability of this particular adult form, rapid identification techniques such as DNA barcoding are quite appealing. Indeed, DNA barcoding via a Basic Local Alignment Search Tool (BLAST) search comparison to reference sequences is often combined with morphological information to confirm the identification of crayfishes (e.g., Barnett et al., 2020; Cabe et al., 2015; Panteleit et al., 2017; Schmidt et al., 2023). Phenotypically unusual specimens have also been identified through DNA barcoding, such as blue‐colored exotic species (Maciaszek et al., 2020). Crayfish species descriptions and delimitation analyses have also used COI barcodes (e.g., Amador et al., 2021; Perkins et al., 2023). Targeted assessments using eDNA for both rare (e.g., Boyd et al., 2020; Quebedeaux et al., 2023) and invasive (e.g., Dougherty et al., 2016; Geerts et al., 2018) species have been successful applications of DNA barcoding, and in the context of crayfish, as have metabarcoding studies (e.g., Drake et al., 2023; Kataoka et al., 2022). Despite the relatively common use of DNA barcoding in crayfish studies, to date, an overall assessment of local and global barcoding gaps across multiple genera has not been conducted.

In this study, our goal was to examine if there are DNA barcoding gaps among crayfish species across a broad suite of genera. Our approach was to (1) establish minimum acceptable sample sizes for assessments of intra‐ and interspecific genetic distance; (2) identify genera that met our sample size requirements in terms of publicly available COI data in the National Center for Biotechnology Information's (NCBI) nucleotide database; (3) quantify “local” barcoding gaps via assessment of the largest intraspecific versus smallest interspecific genetic distances for each congeneric species; (4) quantify “global” barcoding gaps in each genus via mean intraspecific versus mean interspecific genetic distances; and (5) conduct species delimitation to understand the absence of local barcoding gaps.

## 2 METHODS

### 2.1 Units of analysis and dataset development

We considered two alternative units of analysis when determining minimum sample sizes required for inclusion in downstream assessment of DNA barcoding gaps. First, we used the number of different haplotypes per species (*N*_H_), where redundant haplotypes were excluded, which is the approach traditionally used in DNA barcoding studies (e.g., Emery et al., 2009; Lassance et al., 2019). Second, we also considered the total number of COI sequences per species (*N*_S_), where redundant haplotypes were retained. On one hand, N_H_ may inflate intraspecific mean genetic distances given that a very rare divergent haplotype will be weighted equally to all others in the species' gene pool, yet it may seldom be encountered in a DNA barcoding study. On the other hand, *N*_S_ may deflate intraspecific mean genetic distances, particularly if there is a high frequency haplotype and/or gene pool sampling is non‐random with respect to relatedness among individuals (e.g., due to kin clustering). Accordingly, we considered both alternative units of analysis and analyzed them in parallel, to enable assessment of robustness of conclusions about the existence or absence of a barcoding gap. In all cases, we adopted the species‐level specimen identification reported by the authors of sequences uploaded to the NCBI's nucleotide database. Sequences from recently synonymized taxa, or now outdated genus/species names, were reclassified using current crayfish taxonomy (Crandall & de Grave, 2017; Glon et al., 2018, 2022; Taylor et al., 2014). However, we did include as‐yet undescribed species if they met the minimum requirements for the *N*_H_ or *N*_S_ datasets (see below). These undescribed species were present in the genera *Cambarus*, *Cherax*, *Faxonius* (Mathews et al., 2008), and *Tenuibranchiurus* (Dawkins et al., 2017).

To establish minimum *N*_H_ and *N*_S_ values that achieved a balance between reasonably high accuracy of genetic distance estimates without simultaneously rendering many taxa ineligible for inclusion in downstream analyses, we conducted a pilot study. To do this, we used large COI datasets from four exemplar crayfish species (*Faxonius erichsonianus*, *F*. *validus*, *Procambarus paeninsulanus*, and *P*. *spiculifer*) to assess the accuracy of intraspecific COI genetic distance estimation via iterative sub‐sampling of the available data (see below). These species each had >100 COI sequences generated to address population genetic (Barnett et al., 2020) and phylogeographic (Breinholt et al., 2011) questions, and as such, geographic sampling approaches differed (Figure S1). For each of these four species, we first calculated a benchmark mean intraspecific genetic distance using the Kimura two‐parameter (K2P) model (Kimura, 1980) of nucleotide substitution based on all available sequence data, and we treated this as the “true” value. The K2P model was chosen because it was used in the original DNA barcoding studies (Hebert et al., 2003, 2004), and it remains the most widely used model in barcoding studies (e.g., Nishimaki & Sato, 2019). Next, we compared the “true” value to mean K2P distances estimated via 10 replicates of random subsampling along a gradient of increasingly smaller sample sizes (subset sizes were *N* = 3–12 for *N*_H_, and *N* = 5–32 for *N*_S_). The upper limits of this gradient were determined by observing a convergence on the “true” mean K2P distance value followed by plateau with larger subset sizes. To select the minimum *N*_H_ and *N*_S_ values that would be applied in all downstream DNA barcoding gap analyses, we sought a balance between minimizing variance around and departure from the “true” mean K2P distance while also avoiding impractical demands on how extensively sampled intraspecific COI diversity needed to be for a given species.

After determining minimum *N*_H_ and *N*_S_ values, we searched the NCBI nucleotide database for crayfish COI sequences using the following search terms and Boolean operators: “genus name” AND “cytochrome” OR “COI” OR “COX1”. All searches were conducted, and sequences downloaded, between March and December 2022. We aligned sequences from each genus separately using MUSCLE (Edgar, 2004), implemented in MEGA v.7.026 (Kumar et al., 2016). To limit the proportion of missing data, the ends of alignments were trimmed, producing alignment lengths of ≥579 bp (but for some genera we retained a small number of short >200 bp sequences). For quality control, aligned sequences were translated into amino acids to confirm that they had open reading frames. To calculate *N*_H_ for each species, we used the “generate haplotype data file” function in DnaSP v.6.12.03 (Rozas et al., 2017), with the resulting unique haplotypes further checked for unwanted redundancy by generating a pairwise K2P distance matrix in MEGA. *N*_S_ was determined via a simple count of unique NCBI accession numbers. To enable meaningful visualization and assessment of DNA barcoding gaps, for inclusion in this study we required that each genus contained at least four species (all of which satisfied our minimum sample size threshold for *N*_H_ or *N*_S_).

### 2.2 Local and global barcoding gap assessments

For each genus, pairwise intraspecific and interspecific genetic distances were calculated using the K2P model (see above for rationale) in MEGA, assuming uniform rates among sites and treating missing data via pairwise deletion. Evidence for local barcoding gaps was assessed by plotting maximum intraspecific distance versus minimum interspecific distance for each species within a genus, with these datapoints summarized as a scatterplot. If a species had a minimum interspecific distance greater than its maximum intraspecific, it was classified as having a local barcoding gap (Collins & Cruickshank, 2013; Hebert et al., 2004; Meyer & Paulay, 2005). We determined if there was a global barcoding gap by plotting the mean intraspecific versus interspecific distances for each genus (i.e., averaged across its constituent species) as a frequency distribution, summarized as a histogram. For genera with no overlap in mean intraspecific versus interspecific distances (i.e., those with a global barcoding gap), we subsequently determined whether the magnitude of difference satisfied the 10× rule (Hebert et al., 2004). Because there were some differences in species composition of each genus for *N*_H_ versus *N*_S_ datasets (see Section 3), we examined whether this led to significant differences in intraspecific mean distance by directly comparing values in the corresponding *N*_H_ versus *N*_S_ datasets via randomization tests using the “aovp” function in R (R Core Team, 2021) with 10,000 iterations. The same randomization tests were also used to compare mean interspecific K2P distances from corresponding *N*_H_ versus *N*_S_ datasets (see Table S1 and Figures S2 and S3 for justifications of assuming non‐normal distributions).

### 2.3 Phylogenetic tree estimation and species delimitation analyses

To understand why certain species may lack a local barcoding gap, we constructed a phylogenetic tree and performed species delimitation analyses for each genus represented by the *N*_H_ dataset. For computational efficiency, only unique haplotypes (i.e., the *N*_H_ dataset) were analyzed. For each genus in the *N*_H_ dataset, we estimated a maximum‐likelihood phylogenetic tree in IQ‐TREE (Nguyen et al., 2015). The “‐m MFP+MERGE” command was used to automatically find the best nucleotide substitution model for each partition (Chernomor et al., 2016; Kalyaanamoorthy et al., 2017). Following tree inference, node support was assessed using 1000 ultrafast bootstrap replicates, and we interpreted values ≥95% to represent strong support (Hoang et al., 2018).

To distinguish between current taxonomy and potentially different outcomes from species delimitation analyses, herein we refer to taxa that were input into species delimitation analyses as “recognized species”, and the output of these analyses as “delimited species”. For comparison, we performed two types of species delimitation analyses: Assemble Species by Automatic Partitioning (ASAP; Puillandre et al., 2021) and multi‐rate Poisson Tree Process (mPTP; Kapli et al., 2017).

The ASAP method uses ascending hierarchical clustering to determine species partitions using pairwise genetic distances as input. For each partition, the probability of panmixia (*p*‐value) and the relative barcode gap width (W) is calculated, and an ASAP score is produced that is the average ranks of both metrics. Partitions are ranked based on ASAP score, with lower ASAP scores (e.g., lower *p*‐values and larger W) considered as better partitions (Puillandre et al., 2021). All analyses for ASAP were performed using the ASAP web interface (https://bioinfo.mnhn.fr/abi/public/asap/) with the Kimura (K80) substitution model (transition/transversion = 2.0) set to default settings. Because ASAP produces multiple partitions, we based our partition selection on the highest plausible ranking (e.g., not including partitions that consider approximately each sequence as a delimited species). If two partitions were tied in ranking, the partition that delimited fewer species was used for subsequent analysis.

The PTP method models speciation rate directly using the number of nucleotide substitutions, using a non‐ultrametric phylogenetic tree as input (Zhang et al., 2013). Poisson tree process assumes that the number of substitutions between species is significantly higher than the number of substitutions within species, and within‐species branching events are analogous to coalescent events (Zhang et al., 2013). However, this assumes a single exponential distribution for speciation events and coalescent events, respectively, for each species in the phylogeny (Kapli et al., 2017). Thus, we used the multi‐rate PTP model, which fits a distinct exponential distribution for each delimited species, as it tends to produce more accurate numbers of delimited species than PTP (Kapli et al., 2017). All analyses of mPTP were performed using the mPTP web interface (https://mptp.h‐its.org/#/tree) with the default settings.

For ASAP and mPTP separately, outcomes of direct comparison between currently recognized species versus delimited species were classified into one of five possible categories: (1) “perfect taxonomy”, where there was 100% concordance (i.e., a single recognized species was wholly and exclusively contained within a single delimited species); (2) “pure undersplit”, where a recognized species was split into two or more delimited species that were themselves each exclusive groups; (3) “impure undersplit”, which is similar to the former category, except that at least one of the delimited species was not in an exclusive group (i.e., it was clustered together with members of other delimited species); (4) “pure oversplit”, where two or more recognized species were exclusively pooled into a single delimited species; and (5) “impure oversplit”, which is similar to the former category, except that at least one of the recognized species that was pooled with the delimited species also had some members assigned to a different delimited species. Note that the distinction between pure versus impure reflects whether a simple taxonomic change (e.g., naming a new species or synonymizing two species) could versus could not reconcile the difference between current taxonomy and species delimitation outcomes.

## 3 RESULTS

### 3.1 Criteria for inclusion and dataset composition

Our pilot study determined the minimum acceptable sample size was *N*_H_ ≥ 6 and *N*_S_ ≥ 12 (Figures S4 and S5). Given these thresholds, in the resulting genus‐level datasets, some species satisfied one criterion but not the other. Accordingly, *N*_H_ and *N*_S_ datasets were similar but not identical (Table S2). Ultimately, two of the four extant crayfish families were included in our analyses: Parastacidae (three genera) and Cambaridae (five genera) (Table 1). While there were numerous sequences available for Astacidae, no genera had at least four species that met the *N*_H_ or *N*_S_ criteria. Cambaroididae was omitted due to an overall paucity of COI sequence data and a lack of species that were represented. Seven of the eight genera analyzed were represented in both *N*_H_ and *N*_S_ datasets, whereas *Tenuibranchiurus* was only included in the *N*_S_ dataset. Overall, 81 species were represented, with 23 from Parastacidae, and 58 from Cambaridae. Forty‐eight of these species were present in both *N*_H_ and *N*_S_ datasets, with 66 and 63 present in the *N*_H_ and *N*_S_ datasets, respectively.

**TABLE 1:** Summary of local barcoding gaps present in crayfish families and genera, based on each two datasets.

Family	Genus	*N*_H_		*N*_S_		Total examined	
		*N* species	Gap present (%)	*N* species	Gap present (%)	*N* species	Gap present (%)
Cambaridae	*Cambarus*	16	19	11*	18	17	24
Cambaridae	*Creaserinus*	6	0	5	0	6	0
Cambaridae	*Faxonius*	15*	47	15	33	18	50
Cambaridae	*Lacunicambarus*	5	60	8	50	9	56
Cambaridae	*Procambarus*	6	33	7	29	8	25
Parastacidae	*Cherax*	11	55	6	50	11	55
Parastacidae	*Euastacus*	7	43	7	29	8	38
Parastacidae	*Tenuibranchiurus*	–	–	4	25	4	25
	Total	66		63		81	

*Note*: Total examined includes all species examined across both datasets, and includes species that had a local gap present in one dataset, but not the other. Discrepancies between *N*_H_ and *N*_S_ datasets are included, where one species had a local gap present in one dataset (represented by an asterisk) but not the other.

### 3.2 Local and global barcoding gaps

**FIGURE 1:**
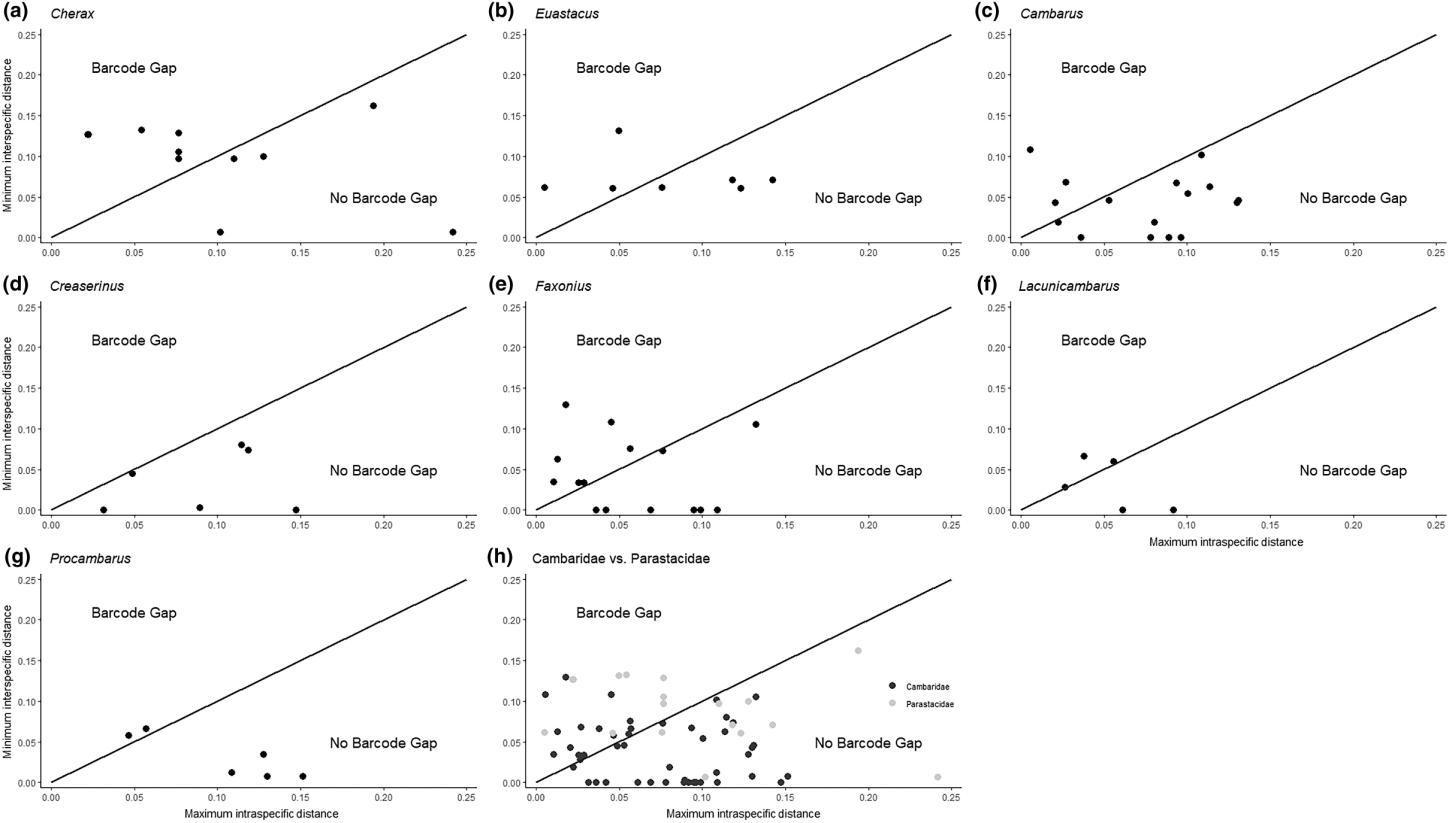
Scatterplots depicting maximum intraspecific K2P distance versus minimum interspecific K2P distance within the genera (a–g) and families (h) examined in the *N*_H_ dataset. Species that fall above the 1:1 line indicate the presence of a local barcoding gap, while species that fall below the line indicate the absence of a local barcoding gap.

**FIGURE 2:**
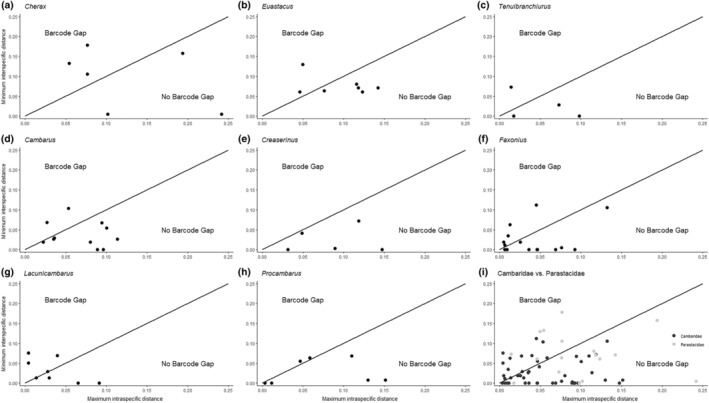
Scatterplots depicting maximum intraspecific K2P distance versus minimum interspecific K2P distance within the genera (a–h) and families (i) examined in the *N*_S_ dataset. Species that fall above the 1:1 line indicate the presence of a local barcoding gap, while species that fall below the line indicate the absence of a local barcoding gap.

Of the 81 species considered, only 30 had a local barcoding gap (Table 1, Table S2, Figures 1 and 2). Local gaps were most abundant in *Lacunicambarus* (Figures 1f and 2g) and *Cherax* (Figures 1a and 2a), with 56% and 55% of species, respectively, in these genera having gaps. Conversely, all *Creaserinus* species lacked local gaps (Table 1, Figures 1d and 2e). This was the only genus where species identification using DNA barcoding was completely unreliable. Regarding overall inferences about the existence versus non‐existence of a local barcoding gap, discrepancies between the *N*_H_ and *N*_S_ datasets were observed in *Cambarus* and *Faxonius*: *C*. *hamulatus* had a local gap present in the *N*_S_ dataset, but absent in the *N*_H_ dataset, whereas *F*. *limosus* had a local gap present in the *N*_H_ dataset but absent in the *N*_S_ dataset (Table S2). Identical mitochondrial COI haplotypes shared between congeneric species, which clearly contribute to the absence of a local barcoding gap, were detected in some members of *Cambarus*, *Creaserinus*, *Faxonius*, *Procambarus*, and *Tenuibranchiurus* (Table S3).

**FIGURE 3:**
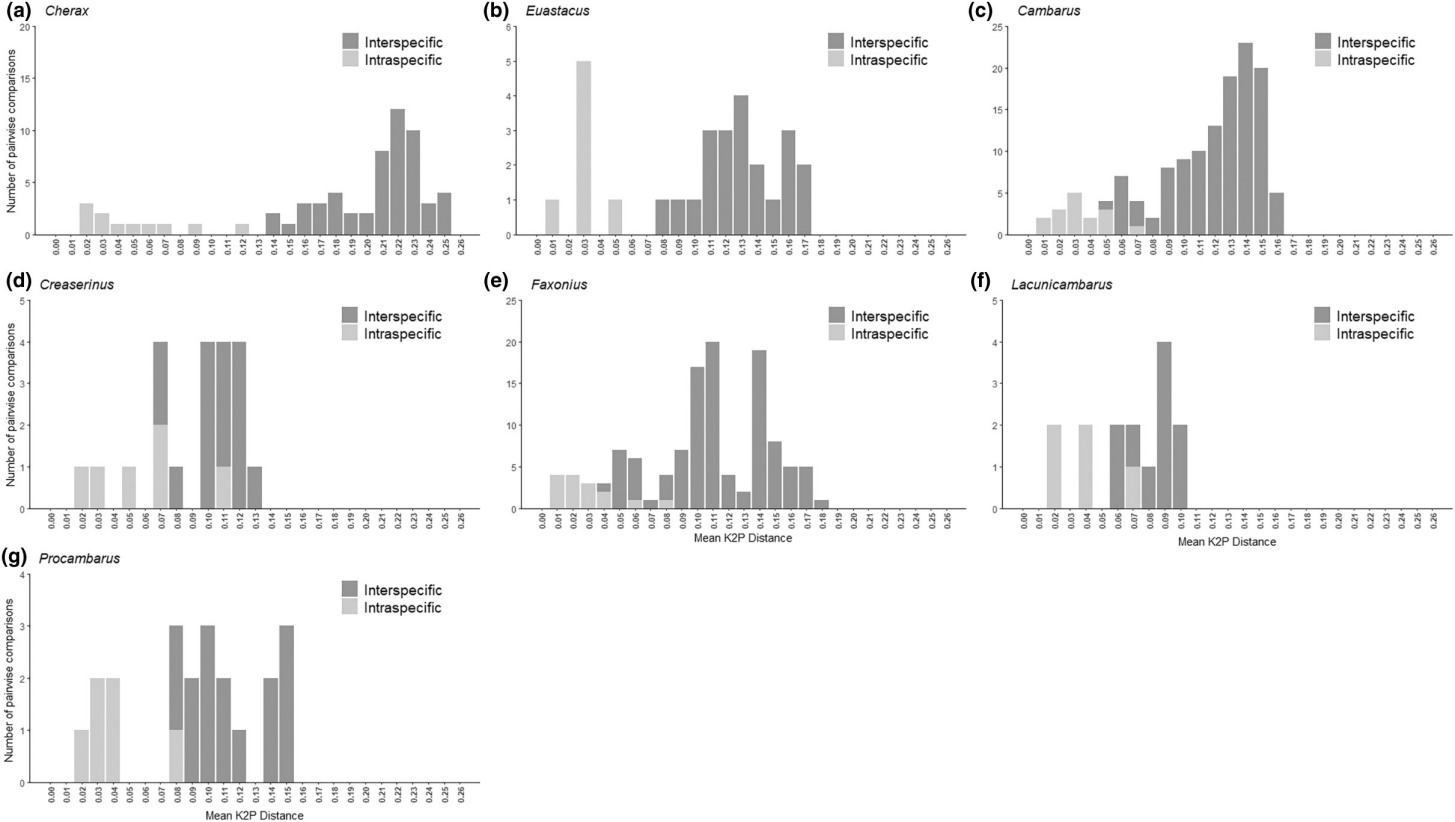
Histograms depicting number of pairwise comparisons of mean intra‐ and interspecific K2P distances within the genera examined in the *N*_H_ dataset. A global barcoding gap is considered present when there is no overlap of intra‐ and interspecific distances (a, b), and is considered absent when there is overlap of intra‐ and interspecific distances (c–g).

**FIGURE 4:**
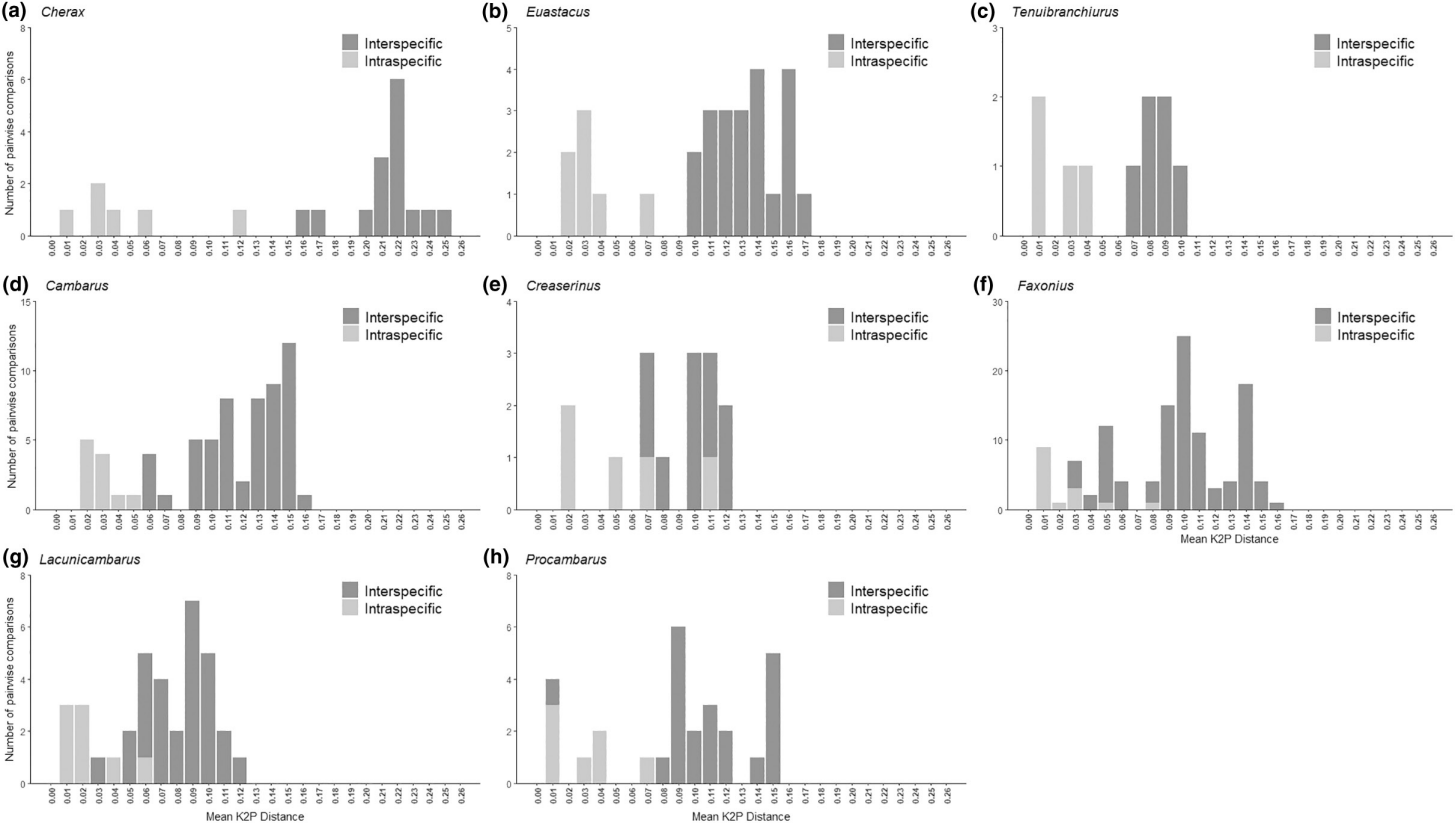
Histograms depicting number of pairwise comparisons of mean intra‐ and interspecific K2P distances within the genera examined in the *N*_S_ dataset. A global barcoding gap is considered present when there is no overlap of intra‐ and interspecific distances (a–d), and is considered absent when there is overlap of intra‐ and interspecific distances (e–h).

Global barcoding gaps were present in four of the eight genera examined: *Cambarus*, *Cherax*, *Euastacus*, and *Tenuibranchiurus* (Table 2, Figures 3 and 4). In these cases, gap thresholds ranged from 4.2× to 5.2×. While global barcoding gaps were present in both *N*_H_ and *N*_S_ datasets for *Cherax*, *Euastacus*, and *Tenuibranchiurus*, different outcomes were obtained for *Cambarus*, depending on the dataset at hand. For this genus, a global gap was absent in the *N*_H_ dataset but present in the *N*_S_ dataset, with the latter representing the only global barcoding gap in any member of Cambaridae (Table 2, Figures 3c and 4d). Interestingly, there were no significant differences in the mean intraspecific K2P distances between corresponding *N*_H_ and *N*_S_ *Cambarus* datasets, nor in their mean interspecific K2P distances (*p* = .237 and .784, respectively; Table 2). The only significant difference between corresponding *N*_H_ and *N*_S_ datasets was for mean interspecific distances among *Faxonius* species (*p* = .007), yet this did not impact inferences regarding the absence of a global barcoding gap in this genus.

**TABLE 2:** Summary of global barcoding gaps present in crayfish families and genera, based on each two datasets.

Family	Genus	*N*_H_			*N*_S_			*p* Value	
		Intra (%)	Inter (%)	Threshold	Intra (%)	Inter (%)	Threshold	Intra	Inter
Cambaridae	*Cambarus*	2.9	11.7	–	2.2	11.6	5.2×	.237	.784
Cambaridae	*Creaserinus*	5.4	9.8	–	4.9	9.2	–	.784	.686
Cambaridae	*Faxonius*	2.3	10.9	–	1.7	9.5	–	.337	**.007**
Cambaridae	*Lacunicambarus*	3.3	7.7	–	1.8	7.7	–	.123	1.000
Cambaridae	*Procambarus*	3.4	11.0	–	2.4	10.4	–	.276	.745
Parastacidae	*Cherax*	4.6	20.5	4.4×	4.4	20.8	4.8×	.882	.765
Parastacidae	*Euastacus*	2.5	12.4	4.9×	3.0	12.8	4.3×	.882	.392
Parastacidae	*Tenuibranchiurus*	–	–	–	2.0	8.1	4.2×	–	–

*Note*: Mean intraspecific (Intra) and interspecific (Inter) K2P distances are reported as % sequence divergence. Gap thresholds, when applicable, indicate the magnitude of a global barcoding gap if it is present. *p* Values are shown for randomization tests comparing mean intraspecific distances between corresponding *N*_H_ versus *N*_S_ datasets, and interspecific distances between corresponding datasets. Bolded *p* values were significant.

### 3.3 Species delimitation analyses

Seven of the eight genera in this study were included in species delimitation analyses (*Tenuibranchiurus* was not represented by a *N*_H_ dataset). While both ASAP and mPTP generally (but not always) had similar conclusions about whether a recognized species should be split apart or lumped together, the total number of delimited species tended to differ between the two methods. Sixty‐six currently recognized species were included in the delimitation analyses, with ASAP delimiting 163 species (~2.5× increase) and mPTP delimiting 124 species (~2×) (Figures [Link], [Link]). Of the 66 recognized species, ASAP indicated that 19 (29%) had perfect taxonomy, 23 (35%) were pure undersplits, 16 (24%) were impure undersplits, and 8 (12%) were impure oversplits (there were no instances of pure oversplit) (Table 3, Figures [Link], [Link]). The mPTP method generated similar results, where 17 (26%) currently recognized species had perfect taxonomy, 24 (36%) were pure undersplits, 15 (23%) were impure undersplits, and 10 (15%) were impure oversplit (again, there were no instances of pure oversplit) (Table 3, Figures [Link], [Link]). For the 66 recognized species, 24 had a local barcoding gap, and of this subset, ASAP and mPTP suggested perfect taxonomy in 79% and 58% of them, respectively (Table 3, Figures [Link], [Link]).

**TABLE 3:** Summary of species delimitation analyses (ASAP and mPTP) results based on the *N*_H_ dataset.

Family	Genus	LBG	# of sp.	ASAP				mPTP			
				PT	PU	IU	IO	PT	PU	IU	IO
Cambaridae	*Cambarus*	3	16	3	6	4	3	3	7	4	2
Cambaridae	*Creaserinus*	0	6	0	3	3	0	1	2	2	1
Cambaridae	*Faxonius*	7	15	5	4	3	3	6	3	3	3
Cambaridae	*Lacunicambarus*	3	5	0	3	2	0	0	1	1	3
Cambaridae	*Procambarus*	2	6	2	0	3	1	1	1	3	1
Parastacidae	*Cherax*	6	11	7	2	1	1	6	3	2	0
Parastacidae	*Euastacus*	3	7	2	5	0	0	0	7	0	0
	Total	24	66	19	23	16	8	17	24	15	10

*Note*: This table reports the following: Number of recognized species with a local barcoding gap present (LBG); total number of recognized species represented in the genus (# of sp.); and the four different types of outcomes that were observed (IO, impure oversplit; IU, impure undersplit; PT, perfect taxonomy; PU, pure undersplit).

## 4 DISCUSSION

In this study, we examined evidence for local and global DNA barcoding gaps in 81 species across eight genera and two families of crayfish. To do this, we used an objective approach to establish minimum acceptable sample sizes for calculating intraspecific and interspecific genetic distances, and we also explored consistency of outcomes across two types of datasets (i.e., *N*_H_, representing unique COI haplotypes only, and *N*_S_, which included redundant haplotypes). Previously, Cabe et al. (2015) employed barcoding approaches to investigate identification success for seven crayfish species across two genera (*Cambarus* and *Faxonius*) in the James River basin, Virginia, USA. Our study expands upon this effort, examining more genera and species, with a broader geographic scope (i.e., spanning Northern and Southern Hemispheres). Below, we discuss local and global barcoding gaps in crayfish, how the units of analysis (*N*_H_ and *N*_S_) may have impacted our results, outcomes of species delimitation analyses, and future research directions.

### 4.1 Local barcoding gaps

Our results showed that DNA barcoding is currently unreliable for species identification in 64%–70% of crayfish species that were examined, depending on the dataset under consideration (Table S2). In *Lacunicambarus*, the genus for which DNA barcoding is most promising, a local barcoding gap was present in 56% of species. Conversely, in *Creaserinus*, the most unreliable genus for DNA barcoding, no local barcoding gaps were present (Table 1). These success rates of COI‐based species identification are lower than those of other invertebrate groups. For example, the presence of local barcoding gaps have been reported for 93% of spider species across 19 genera (Robinson et al., 2009), 98% of aphid species in Pakistan (Ashfaq et al., 2013), and 100% of butterfly species in Pakistan (Naseem et al., 2019). In the present study, several crayfish species that lacked local barcoding gaps owing to unusually high maximum intraspecific K2P distances are believed to be species complexes (e.g., *Cambarus setosus*, *Cherax dispar*, and *Creaserinus fodiens*; Ainscough et al., 2013; Bentley et al., 2010; Graening et al., 2006). When the taxonomy of these and other species is updated, the effectiveness of DNA barcoding for species identification may improve, perhaps considerably.

Non‐native crayfishes have many negative impacts on aquatic ecosystems, including severing aquatic vegetation, displacing native crayfishes, and reducing invertebrate and amphibian abundance (Lodge et al., 2000). DNA barcoding has been recommended for invasive species identification (Armstrong & Ball, 2005). Because crayfish can be difficult to identify, identification through barcoding offers considerable value. However, our analyses showed that several invasive crayfish species (e.g., *Faxonius rusticus*, and *F*. *virilis*; Table S2) lack a local barcoding gap. This finding is alarming, as one of the main applications of DNA barcoding in crayfishes to date has been for confirming invasive species identifications (e.g., Cabe et al., 2015; Filipová et al., 2011). Undoubtedly, the absence of a local barcoding gap in several species included in our study was contributed to by some shared haplotypes with other crayfishes (Table S3). Indeed, hybridization can lead to specimens being incorrectly identified via barcoding (e.g., Rozansky et al., 2021), though misidentifications are another source of error that may also contribute to shared haplotypes. Ultimately, some caution is warranted when using barcoding to identify invasive crayfish species, and as such, dichotomous keys and expert opinion still play an important role in detection and monitoring.

DNA barcoding can be an effective tool for conservation biologists, allowing rapid identification of species (Hebert et al., 2004). This also applies to the aquarium pet trade, where poaching may threaten natural populations of crayfish (Faulkes, 2015). DNA barcoding with species‐specific COI primers has been used to detect rare crayfishes via eDNA (e.g., Cowart et al., 2018; Trujillo‐Gonzalez et al., 2021). While this is likely to continue being reliable for species detection, provided primer design was done with adequate reference sequences, we urge caution when using “universal” invertebrate COI primers such as those used in metabarcoding studies (e.g., Drake et al., 2023). Our results suggest that unreliable results are likely when simply implementing BLAST searches and adopting a sequence similarity threshold for molecular taxonomic identification that has not been vetted for the genus at hand. This is especially important when sister species occur in sympatry (Moritz & Cicero, 2004). Range‐wide sampling of COI haplotypes (e.g., Hurt et al., 2022) coupled with taxonomic work on genera that contain species of conservation concern (e.g., Thoma et al., 2014) is likely to improve the ability of DNA barcoding with universal invertebrate primers to reliably identify crayfish.

### 4.2 Global barcoding gaps

Our analyses showed that global barcoding gaps occurred within only three genera (*Cherax*, *Euastacus*, and *Tenuibranchiurus*) based on both the *N*_H_ and *N*_S_ datasets, and for a fourth genus (*Cambarus*) based on the *N*_S_ dataset alone. However, the magnitudes of these observed gaps were all well below the 10× threshold proposed by Hebert et al. (2004). Indeed, the largest global barcoding gap in the crayfish genera that we examined was approximately only half of what is considered robust (i.e., 4.9× for the *Euastacus N*_H_ dataset, and 5.2× for the *Cambarus N*_S_ dataset; Table 2). Interestingly, the existence of a global barcoding gap in other invertebrate taxa is quite variable. Such gaps have been reported as absent in annelid worms (Kvist, 2016), Cypraeidae gastropods (Meyer & Paulay, 2005), odonate insects (Koroiva & Kvist, 2018), and Palaemonidae shrimp (Robe et al., 2012). Conversely for arachnids, Barrett and Hebert (2005) found no overlap in mean intraspecific and interspecific distances of North American spiders. Čandek and Kuntner (2014) also detected global barcoding gaps in the spider families Tetragnathidae and Lycosidae, of magnitudes that satisfy the proposed “10× rule” (i.e., 11.7× and 20×, respectively). Notwithstanding the widespread absence of global barcoding gaps within the crayfish that we examined, for the genera in which such gaps were detected, we propose that a ~ 5× threshold could act as a useful working hypothesis for species discovery but encourage iteratively reevaluating and updating this as COI data from additional species are made publicly available, and/or as taxonomic updates are published. In the event of an initial identification of two or more clusters of specimens that satisfy a tentative ~5× threshold for crayfish needs to be followed by assessment of the extent to which they satisfy other criteria for consideration as candidate species (i.e., magnitude of overlap in geographic distributions, and existence of congruent differences in any other characters; Padial et al., 2010).

Interestingly, we found that the prevalence of global barcoding gaps differed between families, with members of Parastacidae tending to have these gaps whereas Cambaridae generally did not (Table 2, Figures 3 and 4). These families also have contrasting geographic distributions (i.e., Southern vs. Northern Hemisphere, respectively), and they are thought to have split from a shared common ancestor in the Middle Triassic prior to the breakup of Pangea, around 241 million years ago (Wolfe et al., 2019). In previous phylogenetic analyses, the Southern Hemisphere Parastacidae have long terminal branch lengths, suggesting low recent diversification rates and increased time since speciation (Owen et al., 2015), and divergence as a whole seems much older than Northern Hemisphere crayfish such as Cambaridae (Bracken‐Grissom et al., 2014; Crandall et al., 2000). In contrast, several subclades of North American Cambaridae resulted from recent rapid radiations, and are therefore represented by short branch lengths (Owen et al., 2015). The differences in divergence times and diversification rates could be explained by different biogeographical histories. For instance, in the Southern Hemisphere, Parastacidae may have low recent diversification rates due to formation of the Antarctic Circumpolar Current in the Miocene, and formation of the Antarctic ice sheets and glacial cycles (Owen et al., 2015). Conversely, more rapid diversification in Cambaridae in the Northern Hemisphere may have resulted from niche partitioning (e.g., temperate and cold zones) created by the breakup of Laurasia from Pangea as the landmass moved northward, as has been hypothesized for freshwater insects Corixinae (Ye et al., 2023). These differences in evolutionary history may contribute to low interspecific genetic distances (Cambaridae) or high interspecific genetic distances (i.e., low diversification rates in Parastacidae due to desiccation of Australia; Owen et al., 2015), and thus diminish versus enhance the existence and/or magnitude of global barcoding gaps within genera.

### 4.3 Units of analysis

The *N*_H_ and *N*_S_ datasets usually produced the same overall inferences regarding the presence or absence of local and global barcoding gaps. Also, in nearly all cases, neither mean intraspecific K2P distances between *N*_H_ and *N*_S_ datasets nor their mean interspecific distances, were statistically different. Even in the one instance where there was a significant difference for *Faxonius* in mean interspecific distance, this did not alter our conclusion that a global barcoding gap does not exist in this genus. Taken together, the insensitivity of outcomes to the basic unit of analysis suggests that the commonly used approach of removing redundant haplotypes (e.g., Emery et al., 2009; Garrick et al., 2018; Lassance et al., 2019) prior to employing DNA barcoding may not be necessary. However, there were noteworthy exceptions: the existence of a local barcoding gaps in *C*. *hamulatus* and *F*. *limosus* were dataset‐dependent (Table 1; Table S2), as was the existence of a global barcoding gap in the genus *Cambarus* (Table 2). The underlying cause of these differences may be attributable to different species compositions within the *N*_H_ and *N*_S_ datasets (see Table S2), and/or to the impact of removing versus retaining redundant haplotypes. With respect to the latter, deflated mean intraspecific K2P distances caused by inclusion of redundant haplotypes may create enough separation between mean intraspecific and interspecific genetic distances for a global gap to become apparent. Indeed, establishing a minimum haplotype/sequence sample size requirement for a barcoding study is not trivial. If the requirement is too high, species may be excluded, including important sister species (Meyer & Paulay, 2005), and if too low, intraspecific divergences are then based on a small sample size, which can lead to inaccurate genetic distance estimates. We have presented one approach to striking this balance but encourage further investigation to more fully understand the impact of sample size thresholds on downstream inferences. Indeed, consideration of sampling density in the context of a species' known range (Burgess & Garrick, 2021), and an understanding of whether a species is naturally rare versus newly rare, may be particularly important (Garrick et al., 2015).

### 4.4 Species delimitation analyses

To better understand why some currently recognized species may lack a local barcoding gap, we explicitly considered the potential role of outdated or inadequate taxonomy. To do this, we used two representative DNA‐based species delimitation approaches to find de novo “species” clusters within each genus‐specific dataset and compared the number and composition of these delimited species with the currently recognized ones. While this general approach has been used by others (e.g., Gaytán et al., 2020; Velo‐Antón et al., 2023; Wu et al., 2023), they have tended to focus on evaluating proposed thresholds for flagging candidate new species (i.e., global barcoding gaps). Those studies also had a more tractable geographic scope (cf. our assessment of crayfish from Northern and Southern Hemispheres), which enabled the authors to augment the publicly‐available COI sequence data with targeted field collections of new data. Our species‐specific focus on local barcoding gaps necessitated the creation of a classification scheme to distill the outcomes of comparisons between recognized versus delimited species, so that the outcomes could be readily summarized. We chose to define five categories, which collectively span all possible scenarios. Notwithstanding the limitations of this simplification, it did reveal some interesting insights.

Our species delimitation analyses suggested that of the 66 recognized species examined, 59% (ASAP and mPTP) of them are considered undersplit, while 12% (ASAP) and 15% (mPTP), respectively, are considered oversplit in some way (i.e., either pure or impure). These results mirror the overall absence of local barcoding gaps we observed, suggesting that inadequate taxonomy may be the primary influence on the general unreliability of DNA barcoding for species identification in crayfishes. Indeed, many of the undersplit crayfish species present in our analyses are believed to be species complexes (e.g., *C*. *dispar*, and *C*. *fodiens*; Ainscough et al., 2013; Bentley et al., 2010). Notably, outcomes of our species delimitation analyses indicate that a simple taxonomic change (e.g., naming a new species) will not reconcile the discrepancy, given high numbers of undersplit taxa were classified as “impure”. Oversplitting is another concern, as species that share one or more COI haplotypes (and thus, lack local barcoding gaps) may actually be a single species (e.g., *F*. *virilis*, and *F*. *virilis* complex clade 2; Table S3, Figure S10). Notwithstanding the limitations of single locus species delimitation analyses, we suggest that these approaches may nonetheless be a useful initial step in evaluating the taxonomic status of crayfishes (e.g., Amador et al., 2021; Larson et al., 2016).

Curiously, of the 24 currently recognized species that do have local barcoding gaps, species delimitation analyses suggested that current taxonomy was correct (i.e., “perfect” congruence) in only 58% (mPTP) and 79% (ASAP) of these species. It is possible that the calculation of local barcoding gaps (i.e., highest intraspecific distance vs. lowest interspecific distance) may, owing to the strong influence of outliers, be too stringent for most practical applications of barcoding for species identification. As with species delimitation (e.g., Dupuis et al., 2012), a single locus approach may not be appropriate for species identification in crayfish, and additional loci could be explored. As multilocus datasets become more common in crayfishes (e.g., Glon et al., 2022), we recommend evaluating additional nuclear genes to augment DNA barcoding.

### 4.5 Future directions and conclusions

There are several opportunities for improving on the present study. Ideally, all COI sequences would be drawn only from the Barcode of Life Data System (BOLD) database (Ratnasingham & Hebert, 2007), given its higher submission standards, such as inclusion of precise collection and taxonomic information and high‐quality images of specimens (BOLD, 2023). However, at present, relying solely on crayfish COI sequences from BOLD was intractable, as such stringency would have eliminated most genera from downstream analyses owing to insufficient numbers of sequences, unique haplotypes, and species. Once the BOLD database becomes more well‐populated, reassessment of crayfish DNA barcoding gaps with higher quality data should become possible.

Publicly available COI sequence data are extremely valuable, as they can be repurposed for addressing questions other than what was originally intended by the authors that generated it (Whitlock et al., 2010). Ultimately, our conclusion is that DNA barcoding is currently unreliable for identification of many crayfish species, and we also propose that a global barcoding gap threshold of ~5× may be appropriate for provisional species discovery in these taxa. However, these ideas should be reevaluated in the future as additional data and taxonomic updates become available. Evaluating local and global barcoding gaps in the families Astacidae and Cambaroididae is also recommended, as these families did not meet our sample size requirements. Accordingly, we cannot assume that barcoding will perform poorly for those groups. Nonetheless, DNA barcoding assessments in these families relies on the continued generation of geo‐referenced COI sequence data tied to accessible voucher specimens. This highlights the importance of recent taxonomic updates (e.g., Loughman & Williams, 2021; Perkins et al., 2023; Thoma et al., 2023), investigations into the taxonomic status of crayfishes (e.g., Amador et al., 2021; Bláha et al., 2023; Hildreth et al., 2023), and population genetics studies (e.g., Clay et al., 2020; Hurt et al., 2022) from the Astacology community.

## AUTHOR CONTRIBUTIONS

**Patrick F. Allison Jr:** Conceptualization (lead); data curation (lead); formal analysis (lead); investigation (lead); methodology (lead); writing – original draft (lead). **Emily T. Pickich:** Conceptualization (supporting); data curation (supporting); formal analysis (supporting); investigation (supporting); methodology (supporting); writing – original draft (supporting). **Zanethia C. Barnett:** Conceptualization (supporting); data curation (supporting); formal analysis (supporting); investigation (supporting); methodology (supporting); writing – original draft (supporting). **Ryan C. Garrick:** Conceptualization (supporting); data curation (supporting); formal analysis (supporting); investigation (supporting); methodology (supporting); writing – original draft (supporting).

## CONFLICT OF INTEREST STATEMENT

The authors declare that there is no conflict of interest.

## Supporting information


Data S1

Figure S6

Figure S7

Figure S8

Figure S9

Figure S10

Figure S11

Figure S12

## Data Availability

The data that support the findings of this study are openly available in dryad at https://doi.org/10.5061/dryad.x69p8czqq.
