# Supplementary material for: DNA barcoding is currently unreliable for species identification in most crayfishes
Source: Ecol Evol. 2024 Jul 21;14(7):e70050. doi: 10.1002/ece3.70050 (PMC11260883; doi:10.1002/ece3.70050)
Supplement: Supplementary file 1 — Data S1 [file ECE3-14-e70050-s007.docx]

Allison Jr., P.F., Pickich, E.T., Barnett, Z.C., Garrick, R.C. (2024) DNA Barcoding is Currently Unreliable for Species Identification in Most Crayfish. *Ecology and Evolution*.

**Supplemental Tables**

| **Table S1.** *P* values from Shapiro-Wilk tests for normality in the intra- and interspecific K2P distances in each dataset (N_H_ and N_S_). Bolded values are significant. *Tenuibranchiurus* only had a single dataset (N_S_), and was thus not included in analysis. | | | | | |
| --- | --- | --- | --- | --- | --- |
|  |  |  | |  | |
|  |  | **N_H_** | | **N_S_** | |
| **Family** | **Genus** | **Intra** | **Inter** | **Intra** | **Inter** |
| Cambaridae | *Cambarus* | 0.5382 | **< 0.0001** | 0.0776 | **0.0013** |
| Cambaridae | *Creaserinus* | 0.8010 | 0.1273 | 0.6356 | 0.1990 |
| Cambaridae | *Faxonius* | **0.0136** | **0.0050** | **0.0011** | **0.0002** |
| Cambaridae | *Lacunicambarus* | 0.3226 | 0.2218 | 0.1639 | 0.4037 |
| Cambaridae | *Procambarus* | **0.0192** | 0.0559 | 0.3089 | **0.0130** |
| Parastacidae | *Cherax* | 0.1356 | **0.0024** | 0.1695 | **0.0339** |
| Parastacidae | *Euastacus* | 0.3192 | 0.5545 | 0.0721 | 0.5109 |
| Parastacidae | *Tenuibranchiurus* | -- | -- | -- | -- |

| **Table S2.** List of species represented in the study. Sample size represents the number of unique haplotypes (N_H_) or the number of sequences (N_S_) included in each dataset, respectively. Local barcoding gaps are shown for each species, with discrepancies between N_H_ and N_S_ datasets bolded. | | | | | | |
| --- | --- | --- | --- | --- | --- | --- |
|  |  |  |  | |  | |
|  |  |  | **Sample Size** | | **Local Barcoding Gap** | |
| **Family** | **Species** | **Alignment Length (bp)** | **N_H_** | **N_S_** | **N_H_** | **N_S_** |
| Cambaridae | *Cambarus bartonii* | 654 | 30 | 73 | Absent | Absent |
| Cambaridae | *Cambarus callainus* | 654 | 23 | 23 | Present | Present |
| Cambaridae | *Cambarus deweesae* | 654 | 8 | 13 | Absent | Absent |
| Cambaridae | *Cambarus englishi* | 654 | 14 | 15 | Absent | Absent |
| Cambaridae | *Cambarus guenteri* | 654 | 16 | 16 | Absent | Absent |
| Cambaridae | *Cambarus halli* | 654 | 55 | 56 | Absent | Absent |
| Cambaridae | *Cambarus hamulatus* | 654 | 17 | 19 | **Absent** | **Present** |
| Cambaridae | *Cambarus hazardi* | 654 | 20 | 20 | Absent | Absent |
| Cambaridae | *Cambarus jezerinaci* | 654 | 6 | -- | Absent | -- |
| Cambaridae | *Cambarus jonesi* | 654 | 6 | -- | Absent | -- |
| Cambaridae | *Cambarus longulus* | 654 | 32 | 145 | Absent | Absent |
| Cambaridae | *Cambarus parvoculus* | 654 | 9 | -- | Present | -- |
| Cambaridae | *Cambarus robustus* | 654 | 7 | -- | Absent | -- |
| Cambaridae | *Cambarus sciotensis* | 654 | 10 | 20 | Absent | Absent |
| Cambaridae | *Cambarus setosus* | 654 | 6 | -- | Absent | -- |
| Cambaridae | *Cambarus* sp. 1 | 654 | -- | 18 | -- | Absent |
| Cambaridae | *Cambarus taylori* | 654 | 7 | -- | Present | -- |
| Cambaridae | *Creaserinus brevistylus* | 579 | 12 | 20 | Absent | Absent |
| Cambaridae | *Creaserinus burrisi* | 579 | 28 | 28 | Absent | Absent |
| Cambaridae | *Creaserinus byersi* | 579 | 7 | -- | Absent | -- |
| Cambaridae | *Creaserinus clausus* | 579 | 21 | 23 | Absent | Absent |
| Cambaridae | *Creaserinus fodiens* | 579 | 24 | 28 | Absent | Absent |
| Cambaridae | *Creaserinus limulus* | 579 | 13 | 31 | Absent | Absent |
| Cambaridae | *Faxonius cristavarius* | 626 | 10 | 17 | Present | Present |
| Cambaridae | *Faxonius erichsonianus* | 626 | 43 | 181 | Absent | Absent |
| Cambaridae | *Faxonius etnieri* complex | 626 | 20 | 25 | Absent | Absent |
| Cambaridae | *Faxonius juvenilis* | 626 | 16 | 25 | Absent | Absent |
| Cambaridae | *Faxonius limosus* | 626 | 21 | 110 | **Present** | **Absent** |
| Cambaridae | *Faxonius maletae* | 626 | 8 | -- | Present | -- |
| Cambaridae | *Faxonius obscurus* | 626 | -- | 14 | -- | Present |
| Cambaridae | *Faxonius ozarkae* | 626 | 21 | 42 | Absent | Absent |
| Cambaridae | *Faxonius packardi* | 626 | 9 | -- | Present | -- |
| Cambaridae | *Faxonius punctimanus* | 626 | 11 | 71 | Absent | Absent |
| Cambaridae | *Faxonius rusticus* | 626 | 8 | 13 | Absent | Absent |
| Cambaridae | *Faxonius shoupi* | 626 | 17 | 99 | Present | Present |
| Cambaridae | *Faxonius validus* | 626 | 27 | 149 | Present | Present |
| Cambaridae | *Faxonius virginiensis* | 626 | 11 | -- | Present | -- |
| Cambaridae | *Faxonius virilis* | 626 | 15 | 39 | Absent | Absent |
| Cambaridae | *Faxonius virilis* complex clade 1 | 626 | -- | 20 | -- | Absent |
| Cambaridae | *Faxonius virilis* complex clade 2 | 626 | 8 | 14 | Absent | Absent |
| Cambaridae | *Faxonius virilis* complex clade 3 | 626 | -- | 12 | -- | Present |
| Cambaridae | *Lacunicambarus acanthura* | 585 | 6 | -- | Present | -- |
| Cambaridae | *Lacunicambarus diogenes* | 585 | 14 | 20 | Absent | Absent |
| Cambaridae | *Lacunicambarus erythrodactylus* | 585 | 10 | 12 | Absent | Absent |
| Cambaridae | *Lacunicambarus freudensteini* | 585 | -- | 14 | -- | Present |
| Cambaridae | *Lacunicambarus ludovicianus* | 585 | 13 | 13 | Present | Present |
| Cambaridae | *Lacunicambarus miltus* | 585 | 9 | 24 | Present | Present |
| Cambaridae | *Lacunicambarus mobilensis* | 585 | -- | 19 | -- | Present |
| Cambaridae | *Lacunicambarus nebrascensis* | 585 | -- | 14 | -- | Absent |
| Cambaridae | *Lacunicambarus polychromatus* | 585 | -- | 15 | -- | Absent |
| Cambaridae | *Procambarus acutus* | 630 | 8 | 18 | Absent | Absent |
| Cambaridae | *Procambarus clarkii* | 630 | 65 | 220 | Absent | Absent |
| Cambaridae | *Procambarus fallax* | 630 | -- | 15 | -- | Absent |
| Cambaridae | *Procambarus llamasi* | 630 | 12 | 12 | Absent | Absent |
| Cambaridae | *Procambarus paeninsulanus* | 630 | 47 | 191 | Present | Present |
| Cambaridae | *Procambarus spiculifer* | 630 | 35 | 162 | Present | Present |
| Cambaridae | *Procambarus virginalis* | 630 | -- | 71 | -- | Absent |
| Cambaridae | *Procambarus zonangulus* | 630 | 6 | -- | Absent | -- |
| Parastacidae | *Cherax communis* | 594 | 11 | -- | Absent | -- |
| Parastacidae | *Cherax crassimanus* | 594 | 7 | -- | Absent | -- |
| Parastacidae | *Cherax destructor* | 594 | 9 | 17 | Absent | Absent |
| Parastacidae | *Cherax dispar* | 594 | 65 | 70 | Absent | Absent |
| Parastacidae | *Cherax peknyi* | 594 | 6 | -- | Present | -- |
| Parastacidae | *Cherax preissii* | 594 | 19 | 22 | Absent | Absent |
| Parastacidae | *Cherax quadricarinatus* | 594 | 28 | 154 | Present | Present |
| Parastacidae | *Cherax quinquecarinatus* | 594 | 37 | 86 | Present | Present |
| Parastacidae | *Cherax snowden* | 594 | 6 | -- | Present | -- |
| Parastacidae | *Cherax* sp. n. 2 | 594 | 6 | -- | Present | -- |
| Parastacidae | *Cherax tenuimanus* | 594 | 14 | 16 | Present | Present |
| Parastacidae | *Euastacus balanensis* | 654 | -- | 14 | -- | Absent |
| Parastacidae | *Euastacus bidawalus* | 654 | 7 | 12 | Present | Present |
| Parastacidae | *Euastacus bispinosus* | 654 | 8 | -- | Present | -- |
| Parastacidae | *Euastacus diversus* | 654 | 15 | 35 | Absent | Absent |
| Parastacidae | *Euastacus robertsi* | 654 | 6 | 18 | Present | Present |
| Parastacidae | *Euastacus spinifer* | 654 | 35 | 53 | Absent | Absent |
| Parastacidae | *Euastacus sulcatus* | 654 | 13 | 17 | Absent | Absent |
| Parastacidae | *Euastacus urospinosus* | 654 | 19 | 19 | Absent | Absent |
| Parastacidae | *Tenuibranchiurus glypticus* | 644 | -- | 43 | -- | Absent |
| Parastacidae | *Tenuibranchiurus* sp. 1 | 644 | -- | 16 | -- | Absent |
| Parastacidae | *Tenuibranchiurus* sp. 4 | 644 | -- | 14 | -- | Absent |
| Parastacidae | *Tenuibranchiurus* sp. 5 | 644 | -- | 15 | -- | Present |

| **Table S3.** Summary of 19 crayfish species (both described and undescribed) with shared haplotypes. | | | |
| --- | --- | --- | --- |
|  | |  | |
| **N_H_** | | **N_S_** | |
| **Species 1** | **Species 2** | **Species 1** | **Species 2** |
| *Cambarus halli* | *Cambarus englishi* | *Cambarus halli* | *Cambarus englishi* |
| *Cambarus guenteri* | *Cambarus robustus* | *Creaserinus limulus* | *Creaserinus fodiens* |
| *Creaserinus limulus* | *Creaserinus fodiens* | *Faxonius punctimanus* | *Faxonius virilis* |
| *Faxonius punctimanus* | *Faxonius virilis* | *Faxonius virilis* complex clade 1 | *Faxonius virilis* |
| *Faxonius virilis* complex clade 2 | *Faxonius virilis* | *Faxonius virilis* complex clade 2 | *Faxonius virilis* |
| *Faxonius ozarkae* | *Faxonius punctimanus* | *Faxonius ozarkae* | *Faxonius punctimanus* |
| *Faxonius rusticus* | *Faxonius juvenilis* | *Faxonius rusticus* | *Faxonius juvenilis* |
| *Lacunicambarus erythrodactylus* | *Lacunicambarus diogenes* | *Lacunicambarus erythrodactylus* | *Lacunicambarus diogenes* |
|  |  | *Procambarus fallax* | *Procambarus virginalis* |
|  |  | *Tenuibranchiurus glypticus* | *Tenuibranchiurus* sp. 4 |

**Supplemental Figures**

**
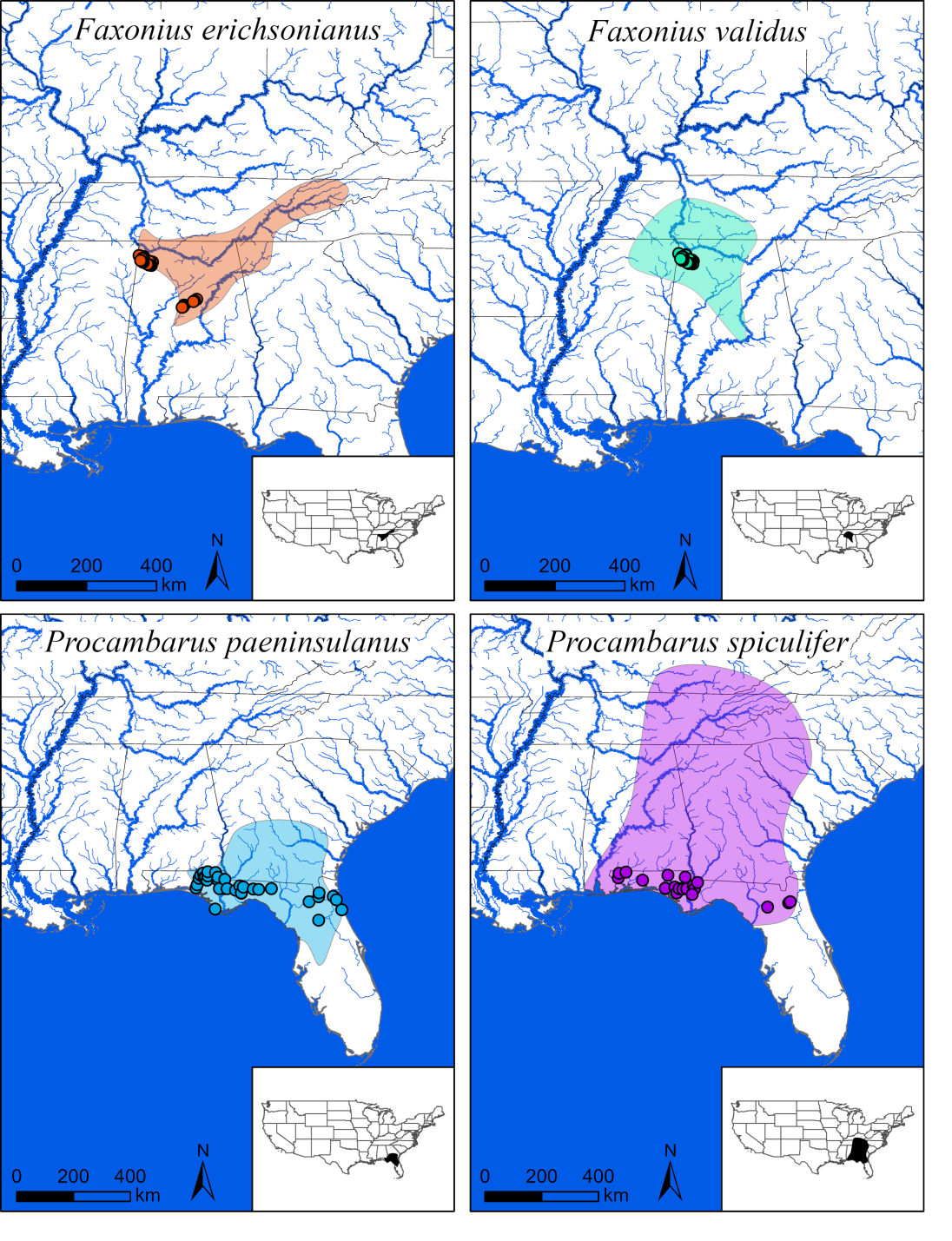
**

**Figure S1.** Distributions (colored backgrounds) of the four exemplar species with sampling localities from Barnett et al. (2020) and Breinholt et al. (2011) used in establishing minimum sample sizes for N_H_ and N_S_ datasets.

**
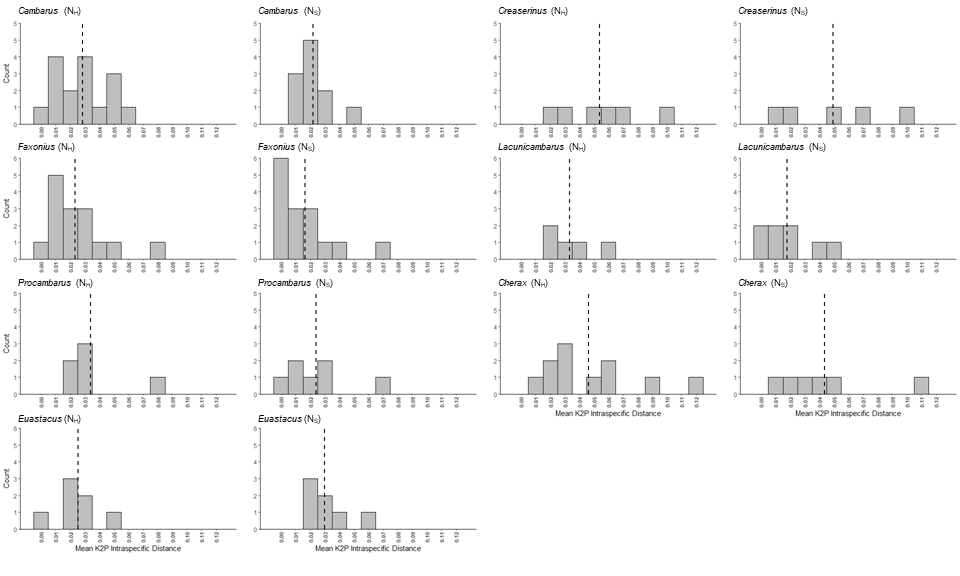
**

**Figure S2.** Histograms of data distribution for the mean intraspecific K2P distances for each genus. The dashed line represents the mean of the mean intraspecific K2P distances.

**
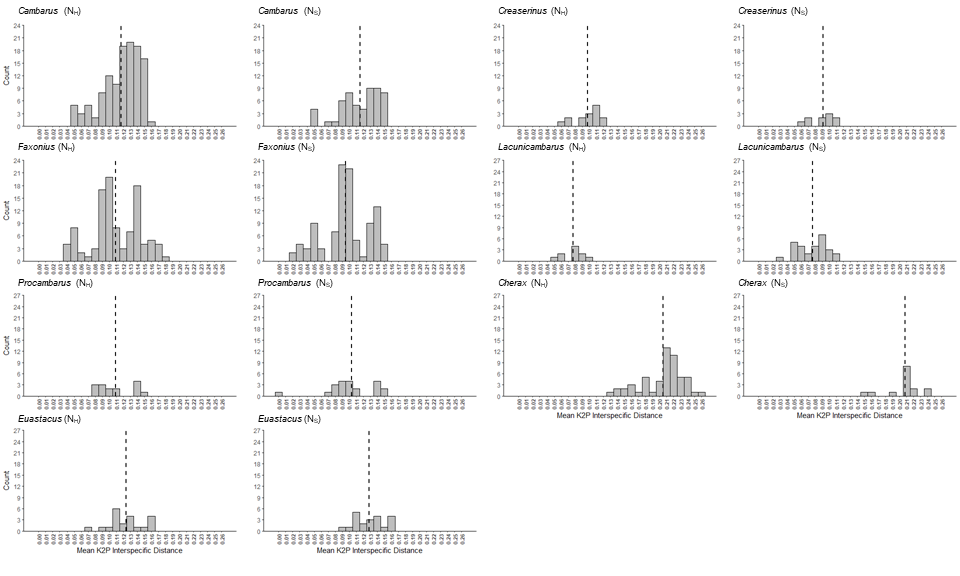
**

**Figure S3.** Histograms of data distribution for the mean interspecific K2P distances for each genus. The dashed line represents the mean of the mean interspecific K2P distances.


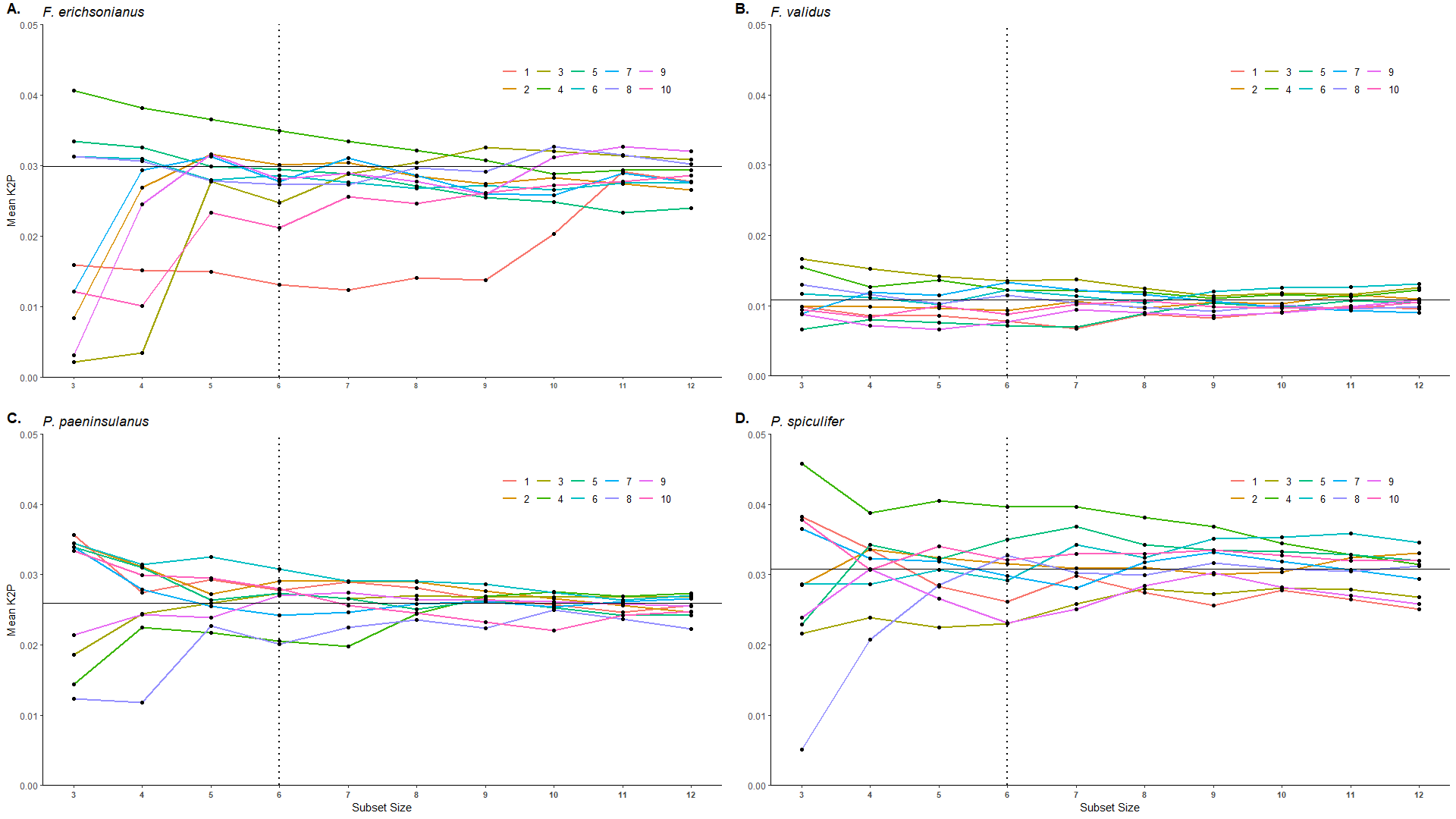


**Figure S4.** Random subsets of 10 replicates of unique haplotype sequences versus mean K2P distance for our four exemplar *Faxonius* and *Procambarus* species. A. and B. are exemplar species from a study that addresses population genetic questions (Barnett et al. 2020), while C. and D. are from a study that addresses phylogeographic questions (Breinholt et al. 2011). We chose six as our minimum sample size for the unique haplotypes dataset (N_H_), represented by the dotted vertical line.


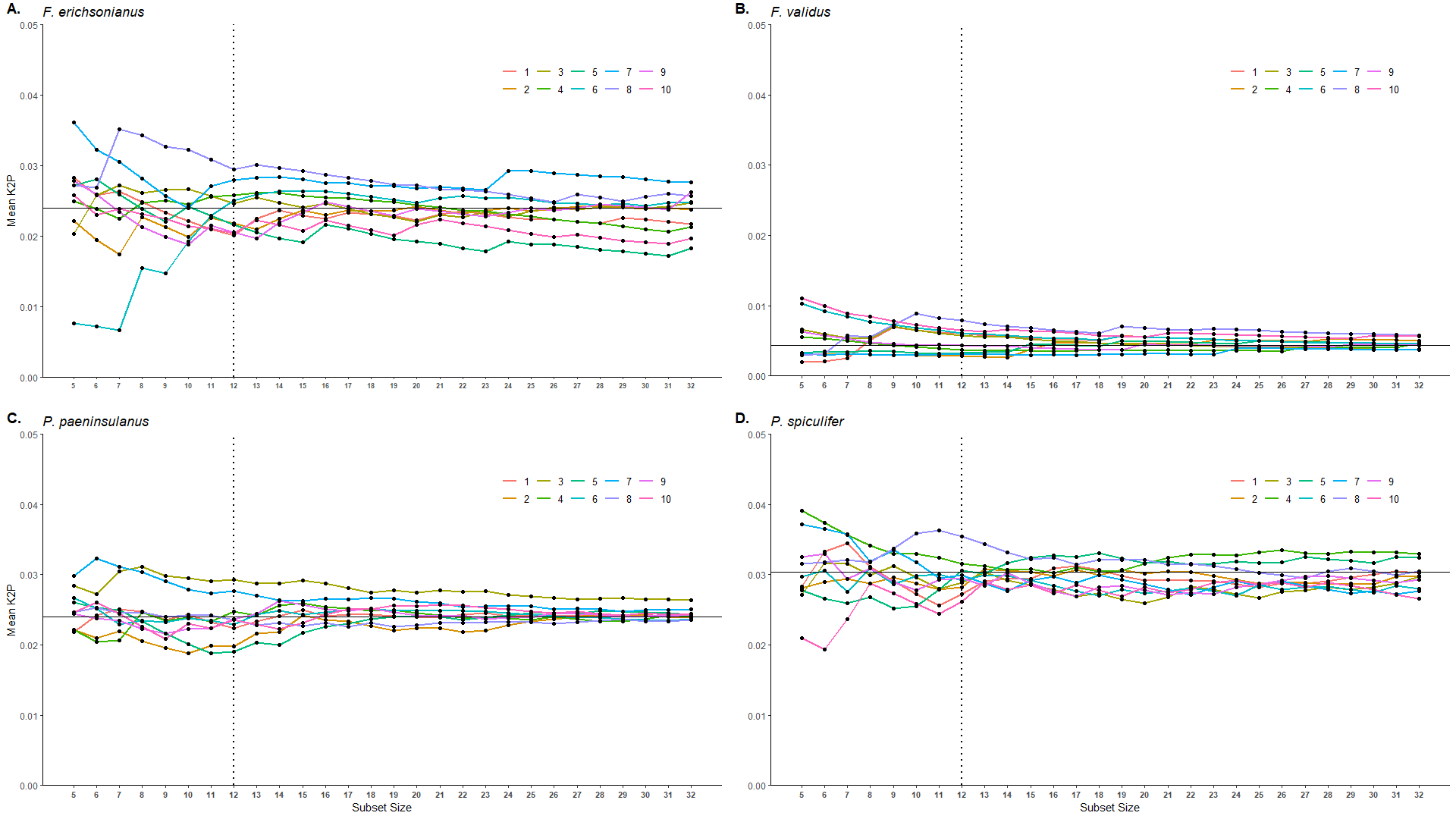


**Figure S5.** Random subsets of 10 replicates of sequences (including redundant haplotypes) versus mean K2P distance for our four exemplar *Faxonius* and *Procambarus* species. A. and B. are exemplar species from a study that addresses population genetic questions (Barnett et al. 2020), while C. and D. are from a study that addresses phylogeographic questions (Breinholt et al. 2011). We chose 12 as our minimum sample size for the sequences dataset (N_S_), represented by the dotted vertical line.
