# Supplementary material for: DNA barcoding is currently unreliable for species identification in most crayfishes
Source: Ecol Evol. 2024 Jul 21;14(7):e70050. doi: 10.1002/ece3.70050 (PMC11260883; doi:10.1002/ece3.70050)
Supplement: Supplementary file 3 — Figure S7 [file ECE3-14-e70050-s001.pdf]

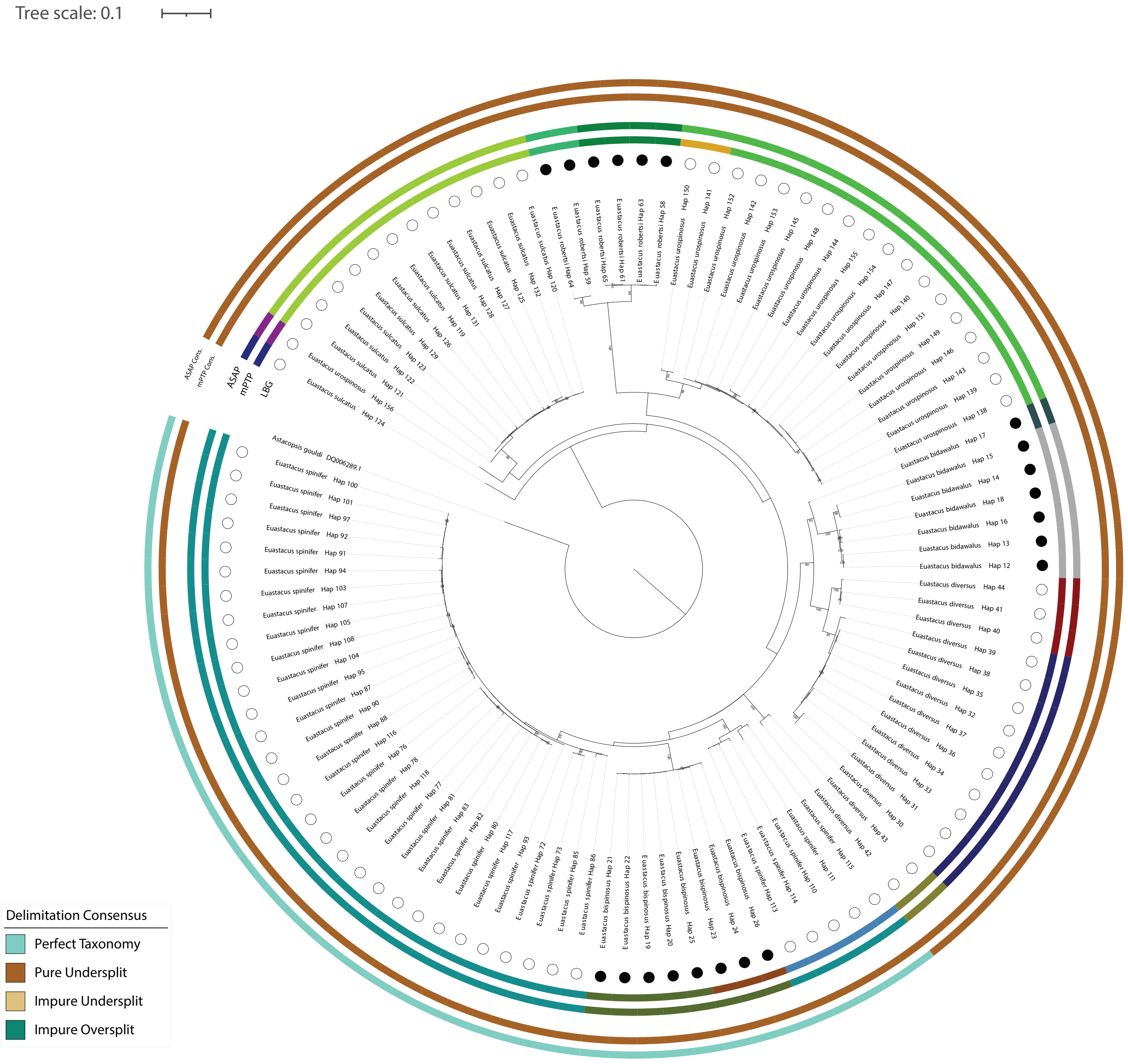

Figure S7. Maximum-likelihood phylogeny estimated from *Euastacus* spp. COI sequences in the N_H dataset. Numbers at nodes indicate ultrafast bootstrap support values ≥ 0.95. Shaded circles represent the presence of a local barcoding gap (LBG). Colored blocks under ASAP and mPTP indicate delimited species groupings for each method. Colored blocks under ASAP Consensus (Cons.) and mPTP Consensus indicate the delimitation consensus category for each method. Created using the Interactive Tree of Life web browser.
